# Task-specific ankle robotics gait training after stroke: a randomized pilot study

**DOI:** 10.1186/s12984-016-0158-1

**Published:** 2016-06-02

**Authors:** Larry W. Forrester, Anindo Roy, Charlene Hafer-Macko, Hermano I. Krebs, Richard F. Macko

**Affiliations:** Maryland Exercise & Robotics Center of Excellence, Veterans Affairs Maryland Health Care System, Geriatrics Research, Education, and Clinical Center, Veterans Affairs Medical Center, Baltimore, MD USA; Department of Neurology, University of Maryland School of Medicine; University Maryland Rehabilitation & Orthopaedics Institute; Maryland Exercise & Robotics Center of Excellence, Veterans Affairs Maryland Health Care System, Baltimore, MD USA; Department of Neurology, University of Maryland School of Medicine; University Maryland Rehabilitation & Orthopaedics Institute; Maryland Exercise & Robotics Center of Excellence, Veterans Affairs Maryland Health Care System; Geriatrics Research, Education, and Clinical Center, Veterans Affairs Medical Center, Baltimore, MD USA; Department of Mechanical Engineering, Massachusetts Institute of Technology, Cambridge, MA USA; Department of Neurology, University of Maryland School of Medicine, Baltimore, MD USA; Department of Physical Medicine and Rehabilitation, Fujita Health University, Toyoake, Aichi Prefecture Japan; Institute of Neuroscience, Newcastle University, Newcastle upon Tyne, UK; Department of Mechanical Sciences and Bioengineering, Osaka University, Osaka, Japan

**Keywords:** Stroke, Hemiparetic gait, Robotics, Locomotor training, Task-specific training

## Abstract

**Background:**

An unsettled question in the use of robotics for post-stroke gait rehabilitation is whether task-specific locomotor training is more effective than targeting individual joint impairments to improve walking function. The paretic ankle is implicated in gait instability and fall risk, but is difficult to therapeutically isolate and refractory to recovery. We hypothesize that in chronic stroke, treadmill-integrated ankle robotics training is more effective to improve gait function than robotics focused on paretic ankle impairments.

**Findings:**

Participants with chronic hemiparetic gait were randomized to either six weeks of treadmill-integrated ankle robotics (*n* = 14) or dose-matched seated ankle robotics (*n* = 12) videogame training. Selected gait measures were collected at baseline, post-training, and six-week retention. Friedman, and Wilcoxon Sign Rank and Fisher’s exact tests evaluated within and between group differences across time, respectively. Six weeks post-training, treadmill robotics proved more effective than seated robotics to increase walking velocity, paretic single support, paretic push-off impulse, and active dorsiflexion range of motion. Treadmill robotics durably improved gait dorsiflexion swing angle leading 6/7 initially requiring ankle braces to self-discarded them, while their unassisted paretic heel-first contacts increased from 44 % to 99.6 %, versus no change in assistive device usage (0/9) following seated robotics.

**Conclusions:**

Treadmill-integrated, but not seated ankle robotics training, durably improves gait biomechanics, reversing foot drop, restoring walking propulsion, and establishing safer foot landing in chronic stroke that may reduce reliance on assistive devices. These findings support a task-specific approach integrating adaptive ankle robotics with locomotor training to optimize mobility recovery.

**Clinical trial identifier:**

NCT01337960. https://clinicaltrials.gov/ct2/show/NCT01337960?term=NCT01337960&rank=1

## Introduction

Stroke is a leading cause of chronic disability, with hemiparetic ankle deficits contributing to impaired gait and balance [1–4]. Current management is limited to either an ankle foot orthosis (AFO) or functional electrical stimulation (FES) that can improve gait velocity, but neither is proven to therapeutically mitigate the underlying ankle neuromotor deficits, except when worn or activated [1, 4–7]. A controversial neuromotor learning question is whether task-specific training or isolated massed-practice across an impaired joint is more effective to improve locomotor function after stroke [8]. This is especially important for ankle which is difficult to therapeutically isolate, and refractory to recovery with more severe deficits such as chronic foot drop.

This randomized study in chronic hemiparetic subjects utilized an impedance-controlled ankle robot (Anklebot: Interactive Motion Technologies, Watertown, MA) with deficit-adjusted adaptive control architecture [9–14] to investigate the hypothesis that 6 weeks Anklebot therapy directly integrated into locomotor treadmill robotic training (TMR) is more effective than matched dose impairment focused seated robotic training (SRT) across the paretic ankle to durably improve unassisted overground gait function and safety.

## Methods

University of Maryland, Baltimore Institutional Review Board and Veterans Affairs Research and Development approved the study (HP-00046304); written informed consent was obtained. Eligibility included adults with mild-moderate severity chronic (>6 months) hemiparetic gait, paretic ankle dorsi-flexor manual muscle test score ≥ 2 (full ROM gravity eliminated) and ≤ 4 (full ROM against gravity, moderate resistance) in dorsiflexion and/or plantarflexion, and capacity to treadmill walk ≥ 0.12 m/sec for 3 min with handrail support. Exclusion criteria included conditions precluding exercise, concurrent physical therapy, and non-stroke mobility disability conditions. Clinical evaluations included screening for dementia, depression, medical and neurological exams, and treadmill exercise stress test. [15] Performance assessments included preferred speed overground walks over an 8-meter instrumented walkway (GaitRite, CIR Systems, Clifton, NJ) and over force plates (Bertec, Columbus, OH), clinical goniometry, paretic ankle motor control measured during unassisted seated, visually-evoked and guided targeting tasks and robot-derived ankle kinematics during unassisted preferred speed treadmill walking (TMR group only) [10–14]. See Table 1 for subject characteristics.

**Table 1:** Subject demographics

Baseline measures (mean ± SE)	Treadmill robotic training (TMR, *n* = 14)	Seated robotic training (SRT, *n* = 12)	*P*-value
Age (years)	59.5 ± 3.6	56.8 ± 3.2	0.88
Sex (male/female)	9 male, 5 female	7 male, 5 female	n/a
Height (m)	1.68 ± 0.03	1.70 ± 0.03	0.81
Weight (kg)	81.5 ± 4.2	85.0 ± 3.7	0.58
Time post-stroke (months)	37.4 ± 10.4	34.0 ± 6.8	0.94
Walking speed (m/s)	0.55 ± 0.06	0.56 ± 0.08	0.94
Berg Balance Scale (0–54)	49.1 ± 1.5	44.3 ± 3.0	0.26
Dynamic Gait Index (0–22)	17.4 ± 0.9	14.4 ± 1.8	0.33
DF AROM (degrees)	1.5 ± 2.4	1.1 ± 5.7	0.87
Assistive device type^a^	8AFO, 8SPC, 1QC, 1RW	7AFO, 5SPC, 3QC, 1RW	n/a

*Abbreviations:* *DF* dorsiflexion, *AROM* active range of motion, *AFO* ankle-foot orthosis, *SPC* single point cane, *QC* quad cane *RW* rolling walker. Wilcoxon Sign Rank *P*-values for between group comparisons. ^a^Note that some subjects used more than one assistive device

Both protocols were initiated by matching task difficulty to baseline ankle deficits, and progressed on performance over 18 sessions (3x weekly; 6 weeks). Each 1-h session of SRT included Anklebot-assisted paretic ankle targeting practice (720 dorsi/plantar-flexion, inversion-eversion repetitions total), with target difficulty progressed (target spacing and frequency increased 38 % and 26 %, respectively) and robotic support decreased, as tolerated [10, 12]. The 1-h TMR sessions aimed for two 15–20-min trials, or as tolerated with rests, at preferred speed (increased from 0.34 to 0.45 m/s and duration from 16 to 37 min), to accumulate a mean number of 889 paretic steps/session, with robotic assistance provided to actuate swing dorsi-flexion or stance plantar-flexion, according to individual gait deficits (i.e. deficit-adjusted) [11, 13, 14]. Level of robotic assistance in early sessions was adjusted to promote foot clearance and push-off, with a tapering of support in the latter sessions to promote autonomy. Robotic assistance was precisely timed to the gait sub-events of interest using insole micro-switches [11, 14].

Outcomes obtained at entry, after 6-weeks training, and 6 weeks post-completion included preferred overground walking speed, paretic limb single support durations, and paretic anterior-posterior propulsive impulses. Seated ankle motor control measures included unassisted volitional targeting speed and accuracy [10, 12], and active range of motion in dorsiflexion. Secondary measures included paretic foot center of pressure (CoP) length and CoP symmetry (paretic-to-nonparetic) during stance [16, 17]. Locomotor learning profile in the TMR group was measured by paretic peak swing angle and heel-first strikes (% footfalls) obtained from robot- and footswitch-measured data during unassisted 1-min treadmill walking before each session [11, 13]. We recorded self-reported changes in utilization of ankle brace and/or assistive device.

Group baseline characteristics were compared using Wilcoxon Sign Rank tests. Fisher’s exact test evaluated between group differences across time points. Non-parametric Friedman tests were performed across all time points, followed by Wilcoxon Sign Rank tests where warranted to evaluate within-group pairwise differences. Two-tail significance was set at 0.05.

## Results

**Fig. 1:**
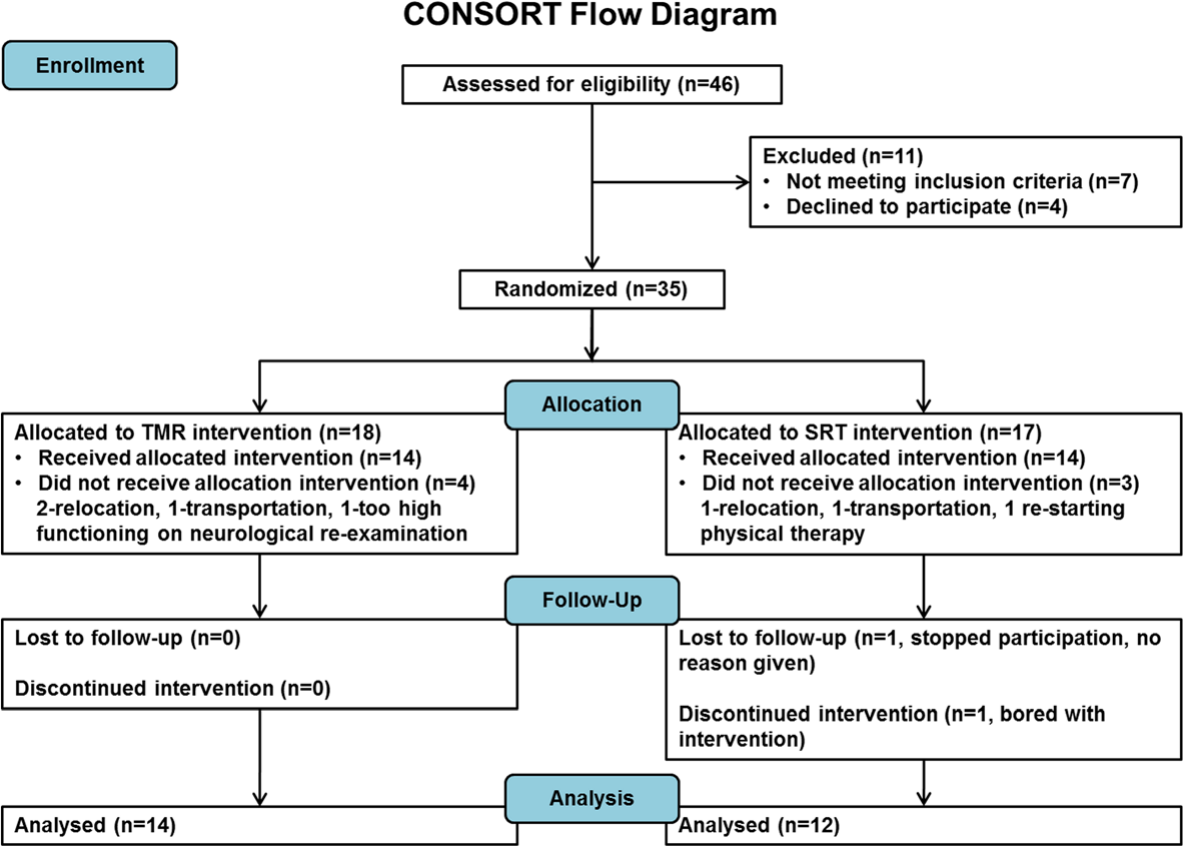
CONSORT flow diagram

Forty-six subjects were screened and thirty-five were randomized; 18 to TMR and 17 to SRT (Fig. 1). Twenty-six completed training for TMR (*n* = 14) and SRT (*n* = 12); attrition was due to relocation (3); transportation (2); physical therapy (1); exclusion on baseline re-test (1); or withdrawal (2). Group demographics did not differ at baseline (Table 1).

After training between group differences in walking velocity showed larger gains for TMR with continued improvement over the six-week retention period (Table 2). SRT did not increase walking velocity at either time-point. TMR increased paretic single support duration, achieving significance at retention testing; SRT elicited no changes. TMR increased paretic propulsive impulse post-training, with further improvement toward 80 % of normal at retention (Table 2) [18], whereas SRT impulses did not change. Paretic foot CoP excursion and CoP symmetry during single support trended toward significance (*P* = 0.10) for TMR at retention. Within group analysis showed improvement in the TMR group for both kinetic measures at retention, whereas SRT showed no changes in dynamic loading during gait.

**Table 2:** Outcomes across testing time points (baseline: PRE, post-testing: POST, follow-up: RETN)

Outcome Variable (mean ± SE)	TMR (*n* = 14)			SRT (*n* = 12)			TMR vs. SRT (*P*-values)	
	PRE	POST	RETN	PRE	POST	RETN	PRE-POST	PRE-RETN
A. Overground gait								
Velocity (cm/sec)	55.5 ± 5.7	58.6 ± 5.5 *P* = 0.20	61.5 ± 5.6 *P* = 0.03	56.0 ± 8.3	56.1 ± 8.5 *P* = 0.70	50.9 ± 7.8 *P* = 0.25	0.24	0.01
Paretic single support (% cycle)	20.7 ± 1.8	21.8 ± 1.8 *P* = 0.12	22.5 ± 1.9 *P* = 0.03	22.0 ± 2.0	21.8 ± 2.0 *P* = 0.89	21.0 ± 2.1 *P* = 0.33	0.23	0.05
Anterior-posterior impulse (Newton-sec.)	−2.5 ± 4.9	9.6 ± 4.1 *P* = 0.009	16.7 ± 6.0 *P* = 0.007	2.1 ± 4.8	0.7 ± 5.1 *P* = 0.72	4.1 ± 5.6 *P* = 0.89	0.11	0.02
Paretic single support center of pressure length (cm)	3.78 ± 0.57	3.81 ± 0.53 *P* = 0.98	4.56 ± 0.59 *P* = 0.009	4.05 ± 1.02	4.30 ± 0.97 *P* = 0.35	4.38 ± 0.94 *P* = 0.48	0.22	0.10
Single support center of pressure symmetry, (paretic-to-nonparetic)	0.52 ± 0.07	0.53 ± 0.08 *P* = 0.64	0.62 ± 0.07 *P* = 0.005	0.60 ± 0.10	0.61 ± 0.09 *P* = 0.70	0.66 ± 0.09 *P* = 0.79	0.21	0.10
B. Ankle motor control								
Ankle targeting speed (deg/sec)	5.4 ± 0.8	5.5 ± 0.5 *P* = 0.93	6.2 ± 0.4 *P* = 0.75	2.5 ± 0.6	4.7 ± 0.4 *P* = 0.005	5.1 ± 0.5 *P* = 0.005	0.07	0.03
Ankle target accuracy (% success)	65.5 ± 7.4	61.9 ± 7.7 *P* = 0.59	70.3 ± 6.7 *P* = 0.31	32.4 ± 7.6	76.0 ± 7.8 *P* = 0.002	70.5 ± 9.4 *P* = 0.005	0.01	0.01
Dorsiflexion active range of motion (deg)	1.5 ± 2.4	12.7 ± 2.5 *P* = 0.004	10.8 ± 2.6 *P* = 0.013	1.1 ± 5.7	5.5 ± 2.1 *P* = 0.53	6.4 ± 1.8 *P* = 0.53	0.11	0.05

*Abbreviations:* *TMR* treadmill robotic training, *SRT* seated robotic training, *SE* standard error. Within group analyses used Wilcoxon Sign Rank test with *P*-values referenced to baseline (PRE) and between groups used Fisher’s exact test

**Fig. 2:**
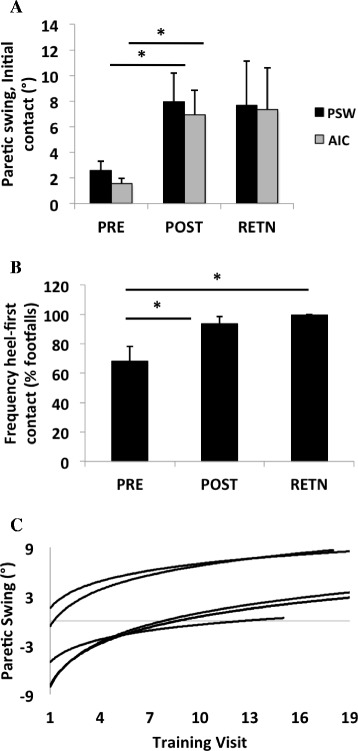
**a** Group data (mean ± SE) from 1-min unassisted treadmill trials at self-selected speed showing paretic peak swing (PSW) and initial contact angles (AIC) at baseline (“PRE”), 6-week post-test (“POST”), and 6-week retention (“RETN”) time points. **b** Group data (mean ± SE) from 1-min unassisted treadmill trials at self-selected speed showing frequency of heel-first ground contact at baseline (“PRE”), 6-week post-test (“POST”), and 6-week retention (“RETN”) time points. **c** Motor learning profiles in unassisted paretic peak swing angle across 18 training sessions from five TMR subjects whose training targeted foot drop. Each profile conforms to a power-law function that is fitted to the peak swing angle averaged across individual steps during a 1-min unassisted trial at self-selected speed, across visits. The profiles are representative of the spectrum of different learning rates in swing clearance

TMR increased peak swing angle and dorsiflexion angle at initial contact during unassisted treadmill walking, with gains sustained at retention (Fig. 2a). These ankle kinematic improvements translated into increased frequency of heel-first ground contacts for most (12/14) TMR subjects (Fig. 2b). Across 18 training sessions, improvement in volitional peak swing angles conformed to a power-law learning model [19], demonstrating emergent autonomous paretic ankle control with different learning rates (Fig. 2c). Improved paretic dorsiflexion active range of motion favored TMR (Table 2), indexed by 7-fold increase post-training and 6-fold at retention; SRT did not differ from baseline at either time point. A majority (5/8) of AFO users in TMR self-reported discarding their AFOs, while 3 others changed to less supportive assistive devices, compared to no changes (0/10) in assistive device use in SRT (*P* < 0.05).

Group differences favored SRT only for changes in seated paretic ankle motor control post-training and at retention testing (Table 2). SRT durably increased mean paretic speed and accuracy of unassisted ankle targeting. TMR produced no gains in seated ankle motor control. Both groups had 100 % training compliance (all 18 sessions attended). Both protocols were well tolerated, with two adverse events including fall while entering their vehicle and an ankle sprain during gait testing.

## Discussion

This is the first randomized study to test the efficacy of impedance-controlled Anklebot with an adaptive control architecture integrated into treadmill training versus impairment-focused seated Anklebot training. The key finding is that TMR, but not SRT, durably improves gait biomechanics and paretic ankle function during independent walking in chronic stroke survivors. TMR progressively increased unassisted paretic swing to normal levels with retained improvements 6 weeks after cessation of training such that a majority of TMR graduates self-discarded their ankle braces. The significant increase in propulsive impulse with TMR to near-normal levels at retention, versus no change in SRT, contributes to ongoing increases in gait velocity after training ended [2, 18]. This unexpected observation that TMR participants continued improving gait measures across the retention phase cannot be fully explained, but anecdotal participant self-reports suggest increased free-living paretic ankle usage. TMR-mediated improvements in paretic leg single support duration, increased paretic foot CoP excursion, and improved spatial symmetry are consistent with improved gait stability [16–18]. Compared to longer-term studies comparing a range of 3–12 months daily AFO versus FES that produced greater gains in gait velocity, the current study reports functional improvements after only 18 training sessions, demonstrating that deficit-adjusted adaptive control Anklebot locomotor training can improve the quality and stability of gait within the time constraints of typical therapy regimens [1, 6, 7].

Durable gains in isolated ankle motor control with SRT is a positive result consistent with our previous studies [10, 12], suggesting that seated Anklebot training may be useful as an adjunct to impairment focused therapies that address isolated ankle neuromotor deficits. The absence of similar gains with TMR also reinforces the notion of training-to-task and highlights its relevance to the evolving field of rehabilitation robotics. Small sample size, brief training duration, and participants with only chronic, mild-moderate gait deficits limit generalizability of these findings. While the two groups experienced similar therapy session durations, the TMR group performed more repetitions per session than for SRT, however both modalities exposed subjects to the same order of magnitude of repetitions (several hundred per day). Gait biomechanics and robotics outcomes were not conducted in a blinded fashion in this pilot study.

## Conclusions

We report that a novel deficit-adjusted approach integrating adaptive ankle robotics into task-specific locomotor training, but not isolated massed practice across the affected joint, improves gait biomechanics and dynamic stability, even years post-stroke. To our knowledge, this is the first therapy, robotic or otherwise, to therapeutically improve functional dorsiflexion and restore impaired push-off during independent walking in chronic stroke enabling individuals to self-reduce reliance on their assistive devices. These results support the overarching hypothesis that in the chronic phase post-stroke, locomotor task-integrated Anklebot training is superior to impairment-focused massed practice across the paretic joint for improving gait function. Larger randomized studies directly comparing TMR to other locomotor rehabilitations approaches are needed to investigate the optimal training paradigm(s) for robotics-assisted rehabilitation across the phases of stroke recovery and for persons with other neurological conditions that include ankle neuro-motor deficits.

## Abbreviations

AFO, ankle foot orthosis; FES, functional electrical stimulation; TMR, locomotor treadmill robotic training; SRT, seated robotic training; CoP, center of pressure.
